# Mapping flow velocity in the human retinal capillary network with pixel intensity cross correlation

**DOI:** 10.1371/journal.pone.0218918

**Published:** 2019-06-25

**Authors:** Phillip Bedggood, Andrew Metha

**Affiliations:** Department of Optometry and Vision Sciences, The University of Melbourne, Melbourne, Australia; Nicolaus Copernicus University, POLAND

## Abstract

We present a new method for determining cellular velocity in the smallest retinal vascular networks as visualized with adaptive optics. The method operates by comparing the intensity profile of each movie pixel with that of every other pixel, after shifting in time by one frame. The time-shifted pixel which most resembles the reference pixel is deemed to be a ‘source’ or ‘destination’ of flow information for that pixel. Velocity in the transverse direction is then calculated by dividing the spatial displacement between the two pixels by the inter-frame period. We call this method pixel intensity cross-correlation, or “PIX”. Here we compare measurements derived from PIX to two other state-of-the-art algorithms (particle image velocimetry and the spatiotemporal kymograph), as well as to manually tracked cell data. The examples chosen highlight the potential of the new algorithm to substantially improve spatial and temporal resolution, resilience to noise and aliasing, and assessment of network flow properties compared with existing methods.

## Introduction

The retina is unique in that it affords direct, non-invasive observation of neural tissue and its associated vascular beds. Recent developments in technology have made possible the visualization of the smallest vessels in the living human retina [1–4]. The smallest vessels within neural tissue offer the greatest resistance to flow and are thought to play a key role in mediating functional changes in flow [5, 6]. Such vessels have been implicated early in the disease process for a variety of conditions including diabetes [7], hypertension [8], stroke [9] and dementia [10]. Recent studies of retinal capillaries using adaptive optics imaging suggest that overt structural damage to capillaries and larger vessels may be preceded by altered capillary flow patterns [11, 12]. Proper characterisation of microvascular flow in the retina may therefore prove important to help elucidate the course of progression for a range of important diseases, and to provide a more sensitive biomarker for the evaluation of potential treatments.

Whilst it is now possible to observe individual blood constituents in the smallest retinal vessels [4], including erythrocytes, leukocytes and even platelets [13], quantifying rate of flow remains challenging due to their small size, low contrast, and high velocity relative to vessel diameter. Existing approaches include manual or part-manual labelling of data [13–15], which are time consuming and may not be amenable to assessment of large numbers of patients in clinical settings. It is possible to “freeze” the raster of a scanning system to rapidly image a given point on a vessel, as long as eye movements are tracked and compensated [13, 16]. This produces highly precise measurements of velocity and other rheological parameters at that position, at the expense of simultaneous collection of data across the vascular network.

This paper will focus on automated approaches to determine velocity, from large numbers of vessels imaged simultaneously in a single video sequence. Previously advocated approaches applicable to this task include particle image velocimetry (“PIV” [4]) and the spatiotemporal kymograph (“STK” [17–21]), which suffer from various limitations summarised below.

PIV involves the division of each spatially-registered image frame into small sub-regions. For each sub-region, the 2D cross-correlation between successive frames is calculated independently. The displacement of the peak of the cross-correlation indicates the distance that blood is presumed to have travelled between frames in a given sub-region. Inherent limitations include:

- Wide sub-regions are desirable for the measurement of fast velocities; whereas
- Small sub-regions are desirable to resolve changes across the network (especially near junctions, crossings, closely packed or sharply turning vessels). Visualising widespread connectivity of flow patterns may be crucial to understanding healthy and pathological flow states [12, 22]; additionally
- Individual velocity estimates are noisy due to being made from single pairs of frames, and somewhat arbitrary rules must therefore be employed to decide which correlations are valid and which are noise [4].

STK involves the creation of a 2D plot where one axis represents spatial position along a vessel (e.g. along the vessel centreline) and the other represents time. Moving cellular material produces a sloped appearance to the plot, with the degree of slope directly proportional to the blood velocity. This approach has been advocated by several groups and currently appears to be the most commonly used for adaptive optics retinal imaging [17–21]. Inherent limitations include:

- The temporal window for analysis should be long to allow the slope of the kymograph to be accurately determined, however, velocity can at times change rapidly due to the cardiac cycle [15, 19] or stochastic variations [4]; furthermore
- An extended length of vessel is desirable for analysis, however, spatial variations in flow have been reported along retinal micro-vessels [4, 18] and are further brought to light here in the Results section. This may require some vessels to be divided into smaller sub-regions, at the cost of accuracy in estimating the slope of the kymograph.
- Vessels must be split into separate segments for analysis, precluding the study of flow at vessel junctions or at abnormal structures such as microaneurysms.
- Single-file flow is generally required so that alternating light and dark regions may be seen (corresponding to plasma or to cells). Thus the method may not be well suited to larger vessels or aberrant flow patterns.
- The method has no way to distinguish between cells of different appearance [18], hence aliasing may be more pronounced than with other approaches.

Our previous work with low-coherence light showed that the microvascular blood column produces an intensity profile in time which carries a (non-speckle) signature that persists for extended distances across the vessel network [18]. This assumedly occurs due to the varying sizes, shapes, separations and arrangements of the blood constituents in capillary flow. The present work proposes that the distance over which this signal propagates following a certain time delay is a source of velocity information. If true this offers a way to redress the limited spatial resolution noted above for the other methods, as velocity can be determined for individual (pairs of) pixels. The temporal resolution would remain high if a time delay of a single frame is found to be sufficient. Such an approach would hold great promise for analysis of microvascular network flow properties e.g. making sense of neurovascular coupling data which are highly suggestive of localised changes in capillary resistance [6]; and identification of areas of defective flow resulting from pathological changes. As will be shown below, a complete spatial map of velocity also offers a powerful tool by which to judge the physiological plausibility of returned velocity measurements. Further, the method need not require cellular material to be separated by plasma gaps (as with the STK method) and does not require noisy pairwise comparisons between frames (as with the PIV method).

## Materials and methods

The general approach advocated here is to make pairwise comparisons of the intensity “trace” (i.e. temporal profile) of each pixel with those for all other pixels in the image sequence, shifted either forwards or backwards in time (e.g. by one frame). The time-shifted pixel that yields the greatest similarity to the reference pixel is found, and the distance of this match from the pixel in question gives the velocity (when divided by the inter-frame period).

The specifics of our data processing pipeline are given below, with some brief commentary regarding each step. The proposed algorithm itself is contained in the section “Core steps in velocimetry calculation”.

### Subjects

Three young, healthy human subjects (age 23–33 years) were recruited from staff and students at the University of Melbourne. All subjects had clear optical media and were adept at maintaining steady fixation. Subjects reported no systemic or ocular disease. Approval was granted by the Human Research Ethics Committee of the University of Melbourne, and subjects were provided with written informed consent prior to experiments. The study conformed with the tenets of the Declaration of Helsinki.

### Image acquisition

Details of our adaptive optics flood illuminated ophthalmoscope system design are as published previously [4]. Data presented here were retrospectively analysed from 3 subjects and were acquired using various imaging wavelengths, frame rates and positions in the retina:

- Imaging wavelength was either 593 ± 25 nm (close to optimal erythrocyte visibility) or 750 ± 25 nm (safer and better tolerated for longer measurements of several seconds in duration).
- Imaging light power at the cornea ranged from 0.33 to 1.3 mW for the sequences presented here, which was 1.0 to 1.3 log units below the maximum permissible exposure according to ANSI standards [23].
- Frame rate was either 200 fps, 300 fps or 400 fps. Our observations regarding aliasing mirror those of Zhang et al [19] who recently concluded that at least 400 fps ought to be used to avoid the majority of aliasing in capillary flow (with the STK method). However, the presentation of lower frame rate data here is instructive as our results suggest that aliasing may be avoided until higher flow velocities with the more robust PIX method.
- Images were acquired over a 1.25° diameter field ranging in eccentricity from ~1–7° from fixation. The best images were generally obtained close to the fovea, whilst lower quality images were obtained remote from the fovea (especially nasally) due largely to scatter from the thicker nerve fibre layer. Example lower quality data is included to demonstrate robustness.

Acquired image sequences were background-subtracted and flat-fielded before being registered via a standard cross-correlation approach as previously described [4]. We corrected only for translational differences and not rotational artifacts in the sequences shown. We believe that the proposed velocimetry method should be applicable to raster-based systems for which intra-frame eye motion-induced distortions become significant, although more complex registration may be required to account for eye movements during frame acquisition [24].

### Pre-processing steps

The following steps are not central to the velocimetry algorithm itself, but were employed to improve processing speed and/or robustness to noise. Each step is applied without any manual guidance.

**Fig 1:**
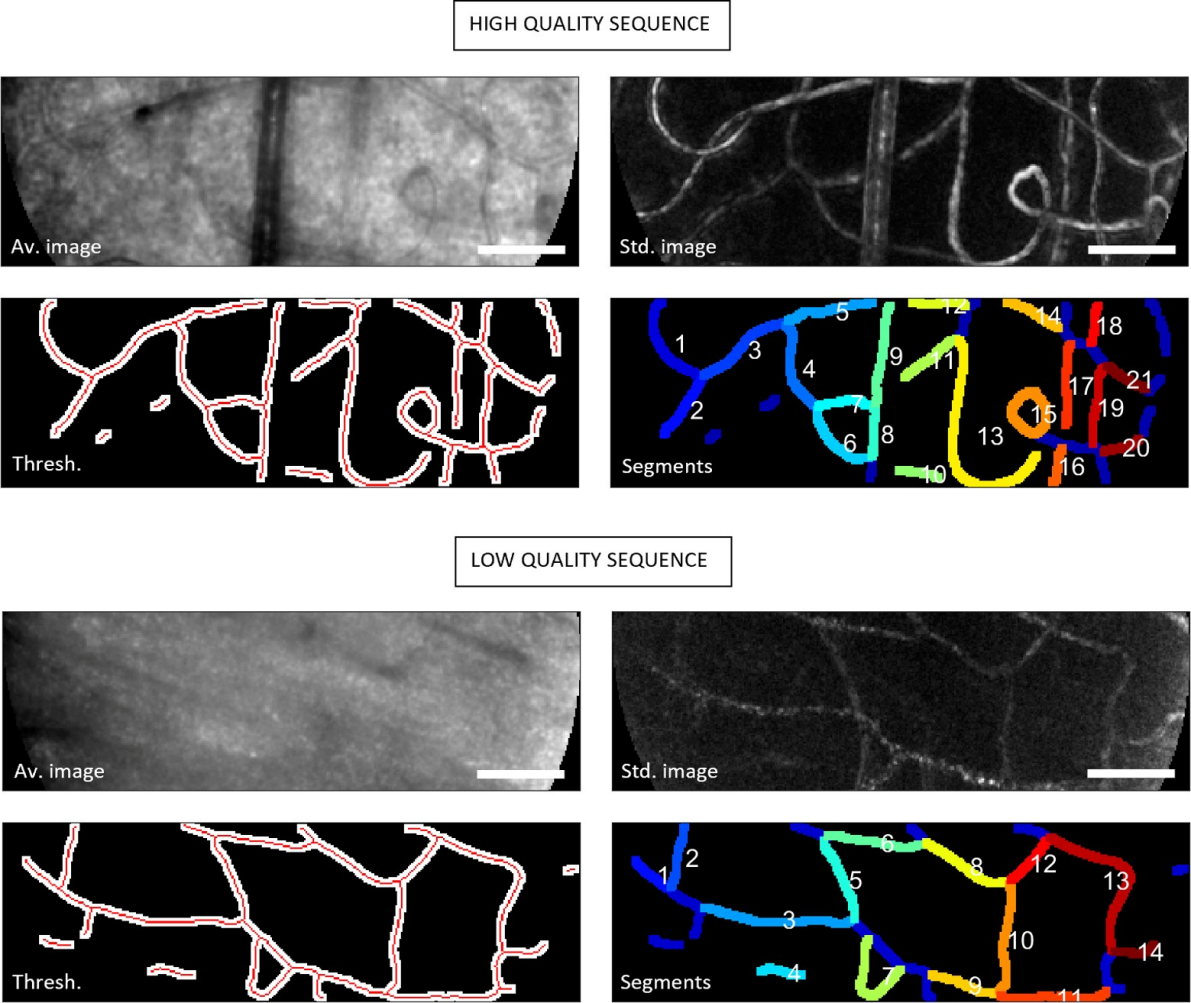
Automated image processing stages used in subsequent analysis. Data from two representative sequences obtained from the same subject at 400 fps with 593 nm light. Top shows higher quality data obtained 2° nasal and 0.5° inferior to fixation. Bottom shows lower quality data obtained 6.5° nasal and 2° inferior to fixation. Top left: Average of 80 frames. Top right: Motion contrast image (standard deviation). Bottom left: White shows binary segmentation, red shows skeletonized vessel segments. Bottom right: Labelled vessel segments. Corresponding raw and filtered data sequences are shown in S1 Video (high signal: noise) and S2 Video (low signal: noise). Scale bars show 50 μm.

1. **Division by frame mean:** Each frame was normalized to have a mean intensity of unity. This was primarily of benefit for sequences acquired with visible light where photoreceptor bleaching is pronounced.
2. **Subtraction of mean intensity profile:** Mean intensity for each pixel was calculated over a rolling 200 ms period (e.g. 60 frames for a 300 fps sequence) and subtracted from the intensity profile. Mean subtraction greatly improves the subjective appearance of flow in our sequences as can be seen in the supplementary videos described below. This is commensurate with improved performance of objective measures also, allowing accurate measurement of velocity in regions of the retina that might otherwise appear too low in vessel contrast to be analysed. A similar strategy was recently described involving division of each pixel by its mean for the entire sequence [15].
3. **Apply temporal filtering.** Fluctuations resulting from various noise sources are further ameliorated by applying a linear de-trend (subtraction of the best straight-line fit to each pixel’s intensity profile), followed by Fourier filtering to remove the very lowest frequency modes of each pixel’s signal (i.e. 2 cycles/sequence). These steps greatly improved robustness to irrelevant variations in intensity e.g. due to bleaching of photoreceptors, or due to misalignment between subject and system pupils resulting from eye/head movement.
4. **Generate motion contrast image.** Motion contrast images were generated by calculation of changes in pixel intensity over time [1, 2]. Fig 1 gives examples of average and standard deviation images (top: a high quality sequence close to the fovea; bottom: a low quality sequence in an area of thick nerve fibre layer; both are from the same subject). S1 Video and S2 Video show the corresponding video sequences.
5. **Generate binary mask of the vasculature.** Motion contrast images were filtered for tube-like structure or “vesselness” [25], using a Gaussian profile with the scale parameter set to 5 μm (on the order of the average retinal capillary diameter). The method can potentially operate at multiple scales if a range of vessel classes are to be considered, which was outside the scope of the present work. The filtered image was automatically thresholded by Otsu’s method [26], with the binary output shown in lower left of each panel in Fig 1. Unless otherwise stated, velocimetry calculations evident in “raw” maps shown below were carried out considering only those pixels lying within the binary mask. This significantly improves processing speed at the expense that the variance in the image sequence must be trusted to demarcate areas of flow.
6. **Divide long sequences into shorter windows.** It is important to choose an appropriate temporal window for velocimetry: too short and the measurements will be noisy, too long and the real velocity will drift during the window due to physiological variations. We chose a period of 100 ms, which is intermediate between our recommendation in the past (150 ms [18]) and the recent recommendation from Gu et al [19] following their survey of a large number of retinal capillaries (75 ms). Successive windows in our analysis were separated by half this period (i.e. a two-fold redundancy in temporal sampling of the sequence).

### Core steps in velocimetry calculation

For the velocity calculation itself, the following steps are carried out for each pixel to be processed within a given temporal window (for example, for each pixel within the image, or for each pixel within a binary segment mask such as shown in Fig 1).

**Fig 2:**
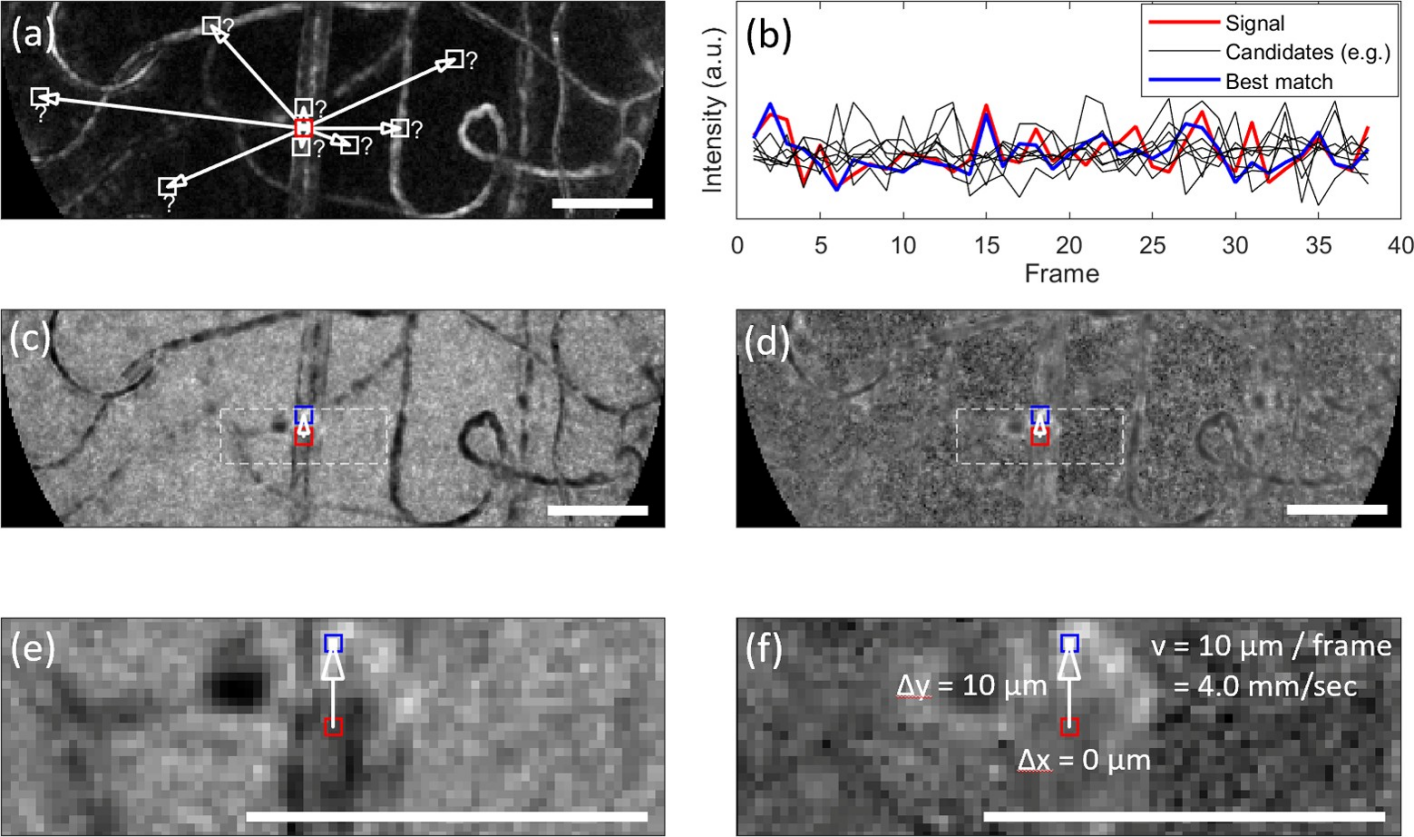
Illustration of velocity calculation for a single pixel. a) Shows the motion contrast image during a 100 ms epoch. The reference pixel whose velocity is to be determined is shown in red. All available image pixels are assayed to determine the best match (white boxes show example candidates); b) shows intensity over time for the reference pixel (red), for the pixel producing the best match after shifting forwards by one frame (blue), and for the other example pixels that were indicated in (a) (black); c) shows the similarity image for the reference pixel (red), which is populated by calculation of root-mean-square (RMS) error between the reference signal and all considered image pixels after shifting by one frame. The brightest pixel (blue) indicates the best match; d) as per (c), but image has been “normalized” by subtracting the standard deviation, which enhances the amplitude of the peak relative to the background; e) as per c), but zoomed according to dashed box shown in (c) for improved visualization; f) as per e), but zoom has been applied to d). The calculation of velocity for this example is overlaid on the image. Scale bars show 50 μm.

1. **Shift the intensity trace both forwards and backwards in time.** Three copies of the intensity profile over time for each pixel are utilised: The original trace, a forward-shifted version (e.g. by one frame) and a backward-shifted version. Example traces are illustrated in Fig 2B (red line shows the intensity profile over time for the reference pixel, other lines show profiles which have been shifted forwards by one frame to assess their similarity to the reference pixel).
2. **Calculate RMS difference of intensity trace from all other pixels.** Pointwise root mean square (RMS) difference is computed between the intensity trace, and the forward- and backward-shifted traces of all pixels. The lowest RMS difference represents the most likely destination or source, respectively, of the information seen by that pixel after the frame lag period (here, one frame) has elapsed. Hence these pixels are taken to be correlated unless proved otherwise (below), and velocity is able to be determined from the displacement between them. To illustrate, the signal which best matched an example reference pixel is plotted in Fig 2B (blue), and can be contrasted against examples of inferior matches (black). The profile plotted in blue corresponds to the brightest pixel on the similarity map (1/RMS) shown in Fig 2C, and the matching inset in Fig 2E.
3. **Remove background structure from RMS difference image.** Background structural information from the vessel network, and probably the photoreceptors, is evident in the raw similarity map but can be removed with a simple procedure. We empirically determined the background to be in direct proportion to the standard deviation (for all pixels other than those corresponding to the interesting part of the image, i.e. the bright peak). We therefore subtracted the standard deviation map from the similarity map, after normalizing their mean and variance to put them on a common scale. The result is shown in Fig 2D and the matching inset in Fig 2F, where the extraneous background information can be seen to have been largely removed. Further elaboration of this point can be found in the Discussion.
4. **Remove non-physiological measurements from further analysis.** Pixels with displacements corresponding to velocities outside the physiologically plausible range* are removed from further analysis (velocity set to NaN). This is a conservative criterion designed to avoid populating the velocity map with unreliable data. We prefer this to the alternative of accepting the best-matching pixel within the physiologically plausible range, despite the fact that the smaller search area would improve computation time. The reason for this is that unreliable pixels (whether not on a vessel, or perhaps on a vessel but of low data quality) are afforded the opportunity to be correlated with a large number of other noisy pixels, making the chances of a strong (spurious) correlation high. Such correlations are statistically likely to be outside the physiologically plausible range since the non-plausible area occupies a much greater fraction of the image. When this occurs, such a pixel will be correctly rejected.
  * Here we focused our attention on capillaries with diameter < 10 μm and so applied a criterion of 4.5 mm/sec, which matches the highest velocity recorded by Zhang et al on their recent high frame-rate survey of many retinal capillaries [19].
5. **Combine forward- and backward-shifted velocities.** To determine the most appropriate single velocity for each pixel, the results of the forward- and back-shifted approaches were averaged. This generally improves robustness to noise compared with use of only forward- or only backward-shifted velocities. In principle, for pixels close to a junction this step could “contaminate” the measured velocity with that from connected segments. For study of flow close to junctions it may be advisable to instead select the direction in time producing the strongest correlation, rather than taking an average.

### Code availability

A MATLAB implementation of the core steps outlined above, together with sample data at 300 fps during a 100 ms epoch, are included in S1 Demonstration. The pre-processing steps described above have already been carried out.

Although the algorithm described here does not in principle require any particular commercial products or operating system to run, S1 Demonstration uses MATLAB code and so requires a MATLAB installation (tested on MATLAB R2017b; only core functions required, i.e. no “toolboxes” necessary).

### Useful post-processing steps

1. **Rejection of unlikely correlations.** In the case of low quality data, the number of spurious correlations increases. This can be largely dealt with by use of a binary mask as described above. An alternate approach is to ascribe some degree of statistical confidence to the measured correlations and reject those which fall below some threshold. Where there exists a measurable physiological relationship between two pixels, a peak is produced in the normalized similarity image (e.g. Fig 2D) that, given the variance encountered in the remainder of the image, would be unlikely to be produced by chance. To formalise this, we adopted a nominal one-tailed p-value threshold of 0.025, divided by the number of “hypotheses” i.e. pixels considered (a Bonferroni correction), and calculated the corresponding Z-score. Potential velocity estimates were rejected if the normalized (i.e. Z-score) similarity image exceeded this value.
2. **Define vessel segments.** For the purposes of a) making comparisons between vessels and velocimetry methods, and b) interpolating data along a vessel, the binary image of the vasculature was further refined by skeletonization, removal of branch points, and rejection of vessel segments less than 25 μm in length. The output of this procedure is shown in red in the bottom left of each panel in Fig 1. The skeleton was then re-dilated by a fixed amount (5 μm for the data presented here) to allow thick vessel segments to be labelled. To avoid overlap between segments, overlapping pixels were flagged and the corresponding skeleton sections removed. The output of this process showing uniquely labelled segments is shown in Fig 1, bottom right of each panel.
3. **Apply “filling” (smoothing and interpolation) to pixels within a vessel segment.** This step produces a common form for the spatial map that facilitates comparison between algorithms. It also improves visualization of spatial gradients in velocity which are apparent along some vessels. We applied 2D median filtering to each pixel, where the raw velocity estimate (NaN in the case of missing data) was replaced by the median of all pixels within 5 μm of the closest valid pixel. Interpolation between segments was not allowed, since we have not made any effort to distinguish vessel junctions from simple crossings. The output of this procedure is presented alongside the raw maps in the Results figures. Although segment averages are well-preserved with this process, all metrics reported/graphed below are nonetheless calculated from the raw data (i.e. smoothed plots are only used as a visual aid).

### Validation against existing methods

The present algorithm was compared to current state-of-the-art methods in retinal capillary velocimetry, i.e. particle image velocimetry (PIV) and spatio-temporal kymograph (STK) methods. Details of our implementation of these approaches have been given previously [4, 18]. The STK method has been independently advocated by, and is currently in favour with, several laboratories [17–21].

To make comparisons between the 3 methods it was useful to attribute velocities to individual vessel segments. As described above, we used a fully automated procedure to segment the vasculature, which will inevitably produce some errors. However, it does afford an objective means by which to compare measurements from different vessels.

PIV and STK algorithms were employed as previously published [4, 18]. Post-processing steps used were identical to those described above. Pre-processing steps were identical other than the following: the temporal filtering step (step #3 in pre-processing above) was not applied to the other methods, as it was found to either make no difference or to produce slightly worse results in some cases; for the PIV approach, to minimize spurious correlations and save processing time we did not compute correlations for regions of interest that did not include the binary vascular mask (this is analogous to step #5 in pre-processing above); the STK approach, being one-dimensional, was conducted on the automatically generated skeleton (e.g. depicted in Fig 1, lower left of each panel), whereas we had previously employed manual tracing of vessel centrelines.

Parameters employed in image processing and velocimetry are compared in Table 1.

**Table 1:** Key parameters used in image processing and velocimetry.

Parameter	PIX	PIV	STK	Units/comments
*Image processing*				
Pixel diameter on retina	1	1	1	μm (binned 2x2 from 0.5 μm)
Epoch duration	100	100	100	ms
Epoch separation	50	50	50	ms
Segment width	5	5	5	μm
Min. vessel length	25	25	25	μm
*Velocimetry*				
Max. velocity	4.5	2.25, 3.75, 4.5	4.5	mm/sec; for PIV, see Ref. 4
Min. velocity		0.3		mm/sec
ROI diam. (small)		12		μm; See Ref. 4
ROI diam. (large)		18, 30, 36		μm; See Ref. 4
ROI separation		6		μm
Limit extent of ST plot			NO	See Ref. 18
Use alternate correlograms*			YES	See Ref. 18
p-value cutoff	0.025			one-tail, Bonferroni corrected

Parameters used in image processing and in velocimetry, for ‘PIX’ (pixel intensity cross-correlation), ‘PIV’ (particle image velocimetry) and ‘STK’ (spatiotemporal kymograph). Shaded cells indicate entries applicable to only one of the velocimetry algorithms.

* To improve robustness of the STK approach, as per our previous work [18] we selected the highest contrast of 3 options: the raw spatiotemporal plot, its 2D spatial correlogram (formed by correlation of the central pixel with other pixels) or its 2D temporal correlogram (correlation of the central frame with all other frames).

### Validation with manually tracked data

In two vessel segments of high image quality, many individual erythrocytes were labelled and tracked throughout longer sequences of 1000 frames at 300 fps (3.3 sec). This corresponds to approximately 3 cardiac cycles in a healthy participant and hence provides a way to compare the ability for each algorithm to track cardiac induced pulsatility, a phenomenon recently reported in the retinal capillaries [15, 19]. Individual cell velocities were grouped into temporal windows corresponding to those used by the automated approaches (see Pre-processing Step 6 above).

## Results

**Fig 3:**
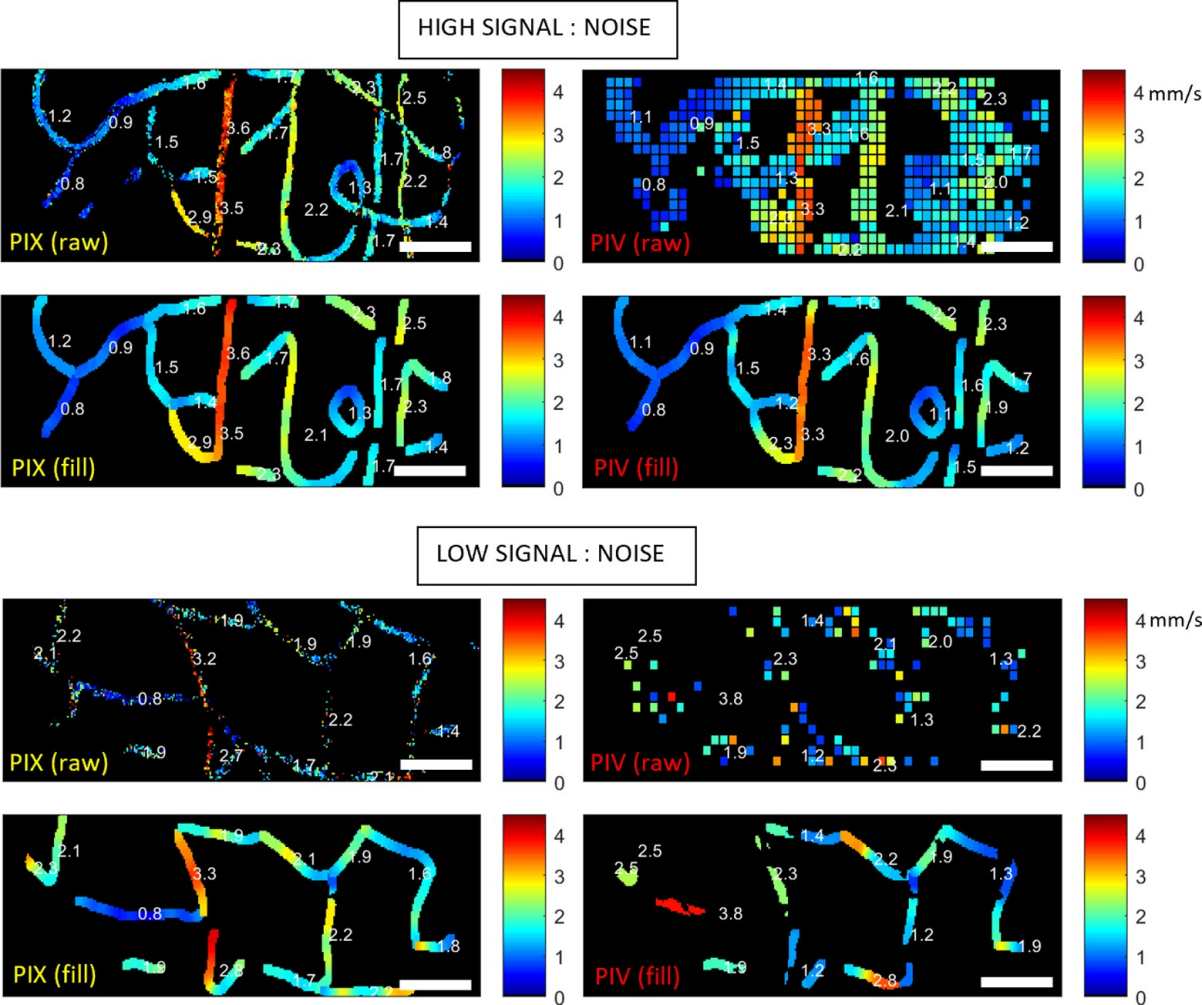
Example spatial maps of flow speed for both PIX and PIV. Panels correspond to the same sequences shown in Fig 1, and in S1 Video and S2 Video. Raw maps are shown at top in each panel, and interpolated maps are shown in the second row. Maps generated by the PIX algorithm are shown left, with comparison maps from PIV shown right. Scale bars show 50 μm.

Example velocity maps generated by the PIX algorithm are shown in Fig 3 (left column), for sequences of high and low quality (top and bottom panels respectively; sequences correspond to those underlying Figs 1 and 2, and can be viewed in S1 Video and S2 Video). For each sequence, both raw data (first row) and filled data (second row) is shown, with the latter facilitating comparison between methods. The right column shows spatial maps generated by the PIV method (maps generated by STK are omitted because they provide limited spatial resolution, with each vessel being coloured uniformly). Comparison of the filled data between PIX and PIV shows strong qualitative agreement for the high quality sequence, but poor agreement for the lower quality sequence. It is possible that alteration of algorithm parameters (Table 1), such as size of sub-regions of interest, could improve the PIV outcome, however, we anticipate little improvement having already made considerable efforts to optimize PIV parameters in previous work [4].

**Fig 4:**
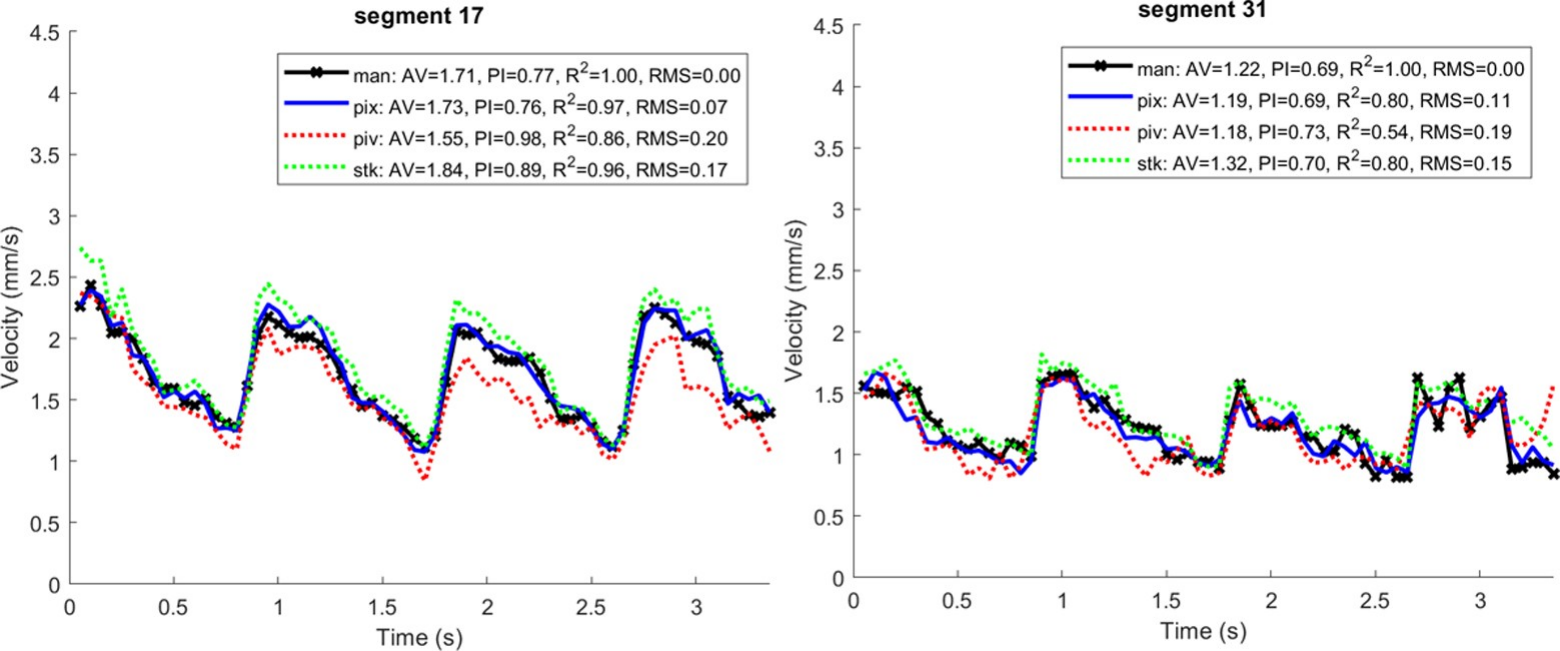
Temporal fidelity in tracking in the face of variability from the cardiac cycle. Sequence was acquired at 300 fps and can be viewed in S3 Video. Two simultaneously imaged segments were tracked manually (left and right panels). Black crosses show manually tracked data, blue shows the output of the proposed PIX method, red shows output for particle image velocimetry (PIV), and green shows output for the spatiotemporal kymograph (STK). The legend shows, from left to right, the average velocity (AV), the pulsatility index (PI, defined as (max-min) / mean), the R^2^ goodness of fit to manually tracked data, and the root-mean-square (RMS) residual error to manually tracked data.

Fig 4 plots data from another subject, for two segments for which a large number of cells have been manually tracked across multiple cardiac cycles at 300 fps (see S3 Video). The segments in question were deemed to have sufficient image quality for reliable manual tracking of each cell and so represent a “best case” scenario for tracking of temporal variations. The mean velocity, pulsatility index (PI; amplitude normalised to mean), quality of correlation (R^2^) and residual error (root-mean-square) are shown for ease of comparison. Some discrepancy is expected between areal methods such as manual tracking, PIV and PIX, compared with the STK approach which determines velocity tangential to a presumed vessel centreline. There was strong agreement between the new method, the other automated methods and the manual tracking data, demonstrating proof of principle of the algorithm. The faithful rendering of large variations in velocity with a period ~1 sec demonstrate the ability of the new method to track slow change in time, on the order of the cardiac cycle.

**Fig 5:**
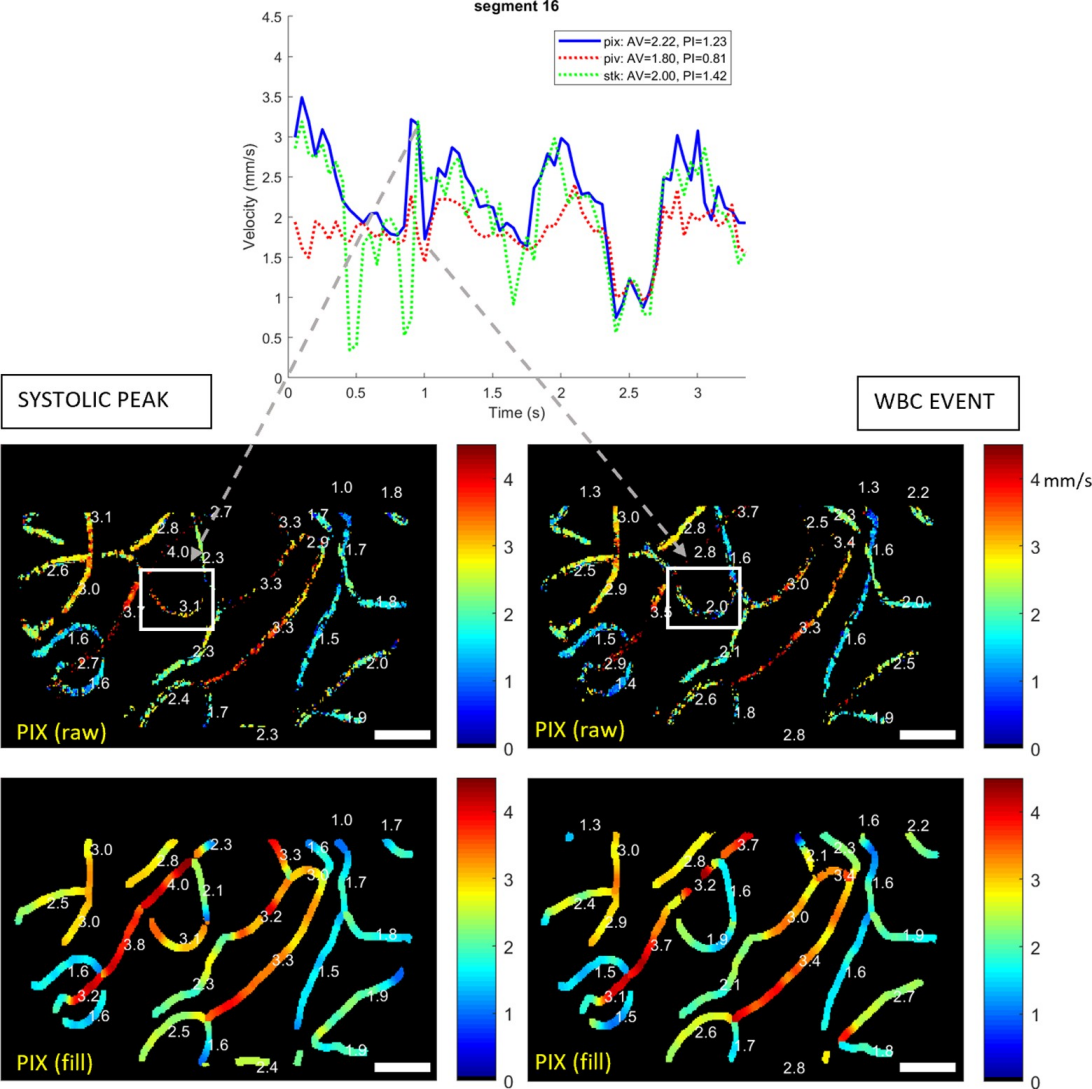
Influence of a white blood cell on capillary velocity. Top: Velocity trace for one segment showing long-term and short-term fluctuations in velocity captured with the PIX algorithm. Arrows indicate a rapid drop from the presumed systolic peak at ~ 3 mm/sec to ~2 mm/sec, which occurs due to passage of a white blood cell. Bottom: PIX velocity maps corresponding to the time points indicated by arrows. White boxes: the vessel segment plotted whose average velocity is plotted in top. Raw and mean-subtracted sequence can be viewed in S3 Video, and evolution of velocity maps in time can be viewed in S4 Video. White boxes indicate the vessel whose average velocity is plotted top. The legend shows, from left to right, the average velocity (AV), the pulsatility index (PI, defined as (max-min) / mean), the R^2^ goodness of fit to manually tracked data, and the root-mean-square (RMS) residual error to manually tracked data. Scale bars show 50 μm.

Fig 5 (top) presents data which highlights some advantages of the new method. The plot corresponds to another, faster segment from the same sequence as Fig 4. Relative to the phase of the presumed cardiac waveform that was seen in Fig 4, we observe the following discrepancies:

- PIX produces a sudden trough just after the systolic peak at approx. 1.0 sec
- STK shows sudden troughs in the diastolic phase at approx. 0.45, 0.85 and 1.65 sec
- PIV largely fails to imitate the cardiac waveform faithfully

Inspection of S3 Video reveals subjective aliasing of flow at certain time points; for example, seeming reversal of flow at each of the sudden troughs reported above for the STK algorithm. Aliasing likely explains the poor performance of the PIV algorithm as well, which similarly has no way to easily handle multiple particles appearing in the same ROI [4].

It might be concluded that the vessel is not able to be easily studied, however, PIX produced a convincing cardiac waveform albeit with the large trough noted above. Further inspection reveals the trough to be a physiological event, not an error. At frames 296–326 in S3 Video a period of stasis and cell accumulation is evident, associated with the passage of a white blood cell. This slows the flow while the cell and associated traffic pass, before the segment “catches up” to a level more commensurate with the systolic pulse wave. The temporal windows just before and during this event are contrasted in the PIX velocity map of Fig 5. Comparison of these maps for the vessel in question (white box) show an increase in vessel diameter and a “sheathing” appearance associated with the passage of the white blood cell. This can be further appreciated in S4 Video which shows the evolution of the PIX spatial map in time (played at one-quarter of real-time speed). The faithful rendering of transient changes such as this demonstrates the potential for PIX to offer superior short-term tracking of flow. The faithful reproduction of the cardiac cycle in this segment (whose phase, as expected, matches the easier-tracked segments from Fig 4) further suggests that the method is resilient to aliasing which affects not only the other two methods, but the subjective impression of flow when watching the movie (S3 Video).

To determine whether a velocity map is physiologically plausible, it is useful to consider what reasonable constraints should be applied. We would propose that:

- Reported velocities must lie within the physiological range
- Variations over time should smoothly follow the expected pattern of the cardiac cycle, unless there is some clear explanation for the departure (e.g. passage of a white blood cell as noted above)
- Velocity within a segment should be either relatively homogenous, or perhaps display gentle gradients, but should not oscillate dramatically.
- In some cases sharp changes in velocity should be evident, where cells pass from one segment into another segment which has a markedly different velocity.

**Fig 6:**
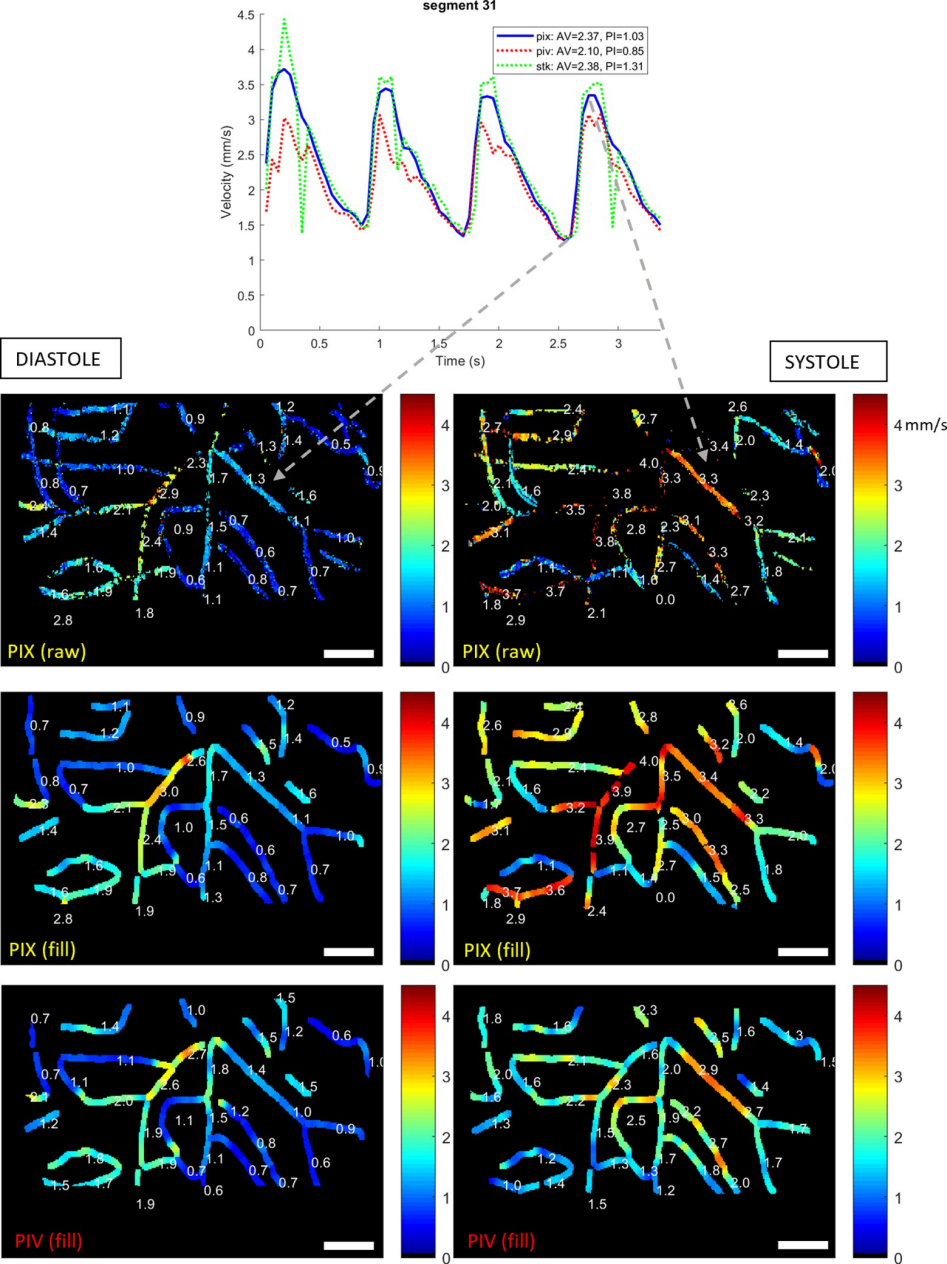
Spatial velocity maps comparing performance for slow and fast flow. Top: velocity trace used to infer phase of the cardiac cycle. Left column: presumed diastole. Right column: presumed systole. Sequence was acquired at 300 fps and can be viewed in S5 Video. Top plot confirms the phase of the pulse wave. Second row: PIX (raw). Third row: PIX (filled). Bottom row: PIV (filled). Physiological consistency across the network appears preserved in this sequence during systole with PIX, but not with PIV, which is unable to track the faster systolic flow. The legend shows, from left to right, the average velocity (AV), the pulsatility index (PI, defined as (max-min) / mean), the R^2^ goodness of fit to manually tracked data, and the root-mean-square (RMS) residual error to manually tracked data. Scale bars show 50 μm.

The data shown in Figs 3, 4 and 5 (and the evolution shown in S4 Video) offer compelling evidence that the PIX algorithm does produce physiologically plausible output. Fig 6 builds on this by showing data from another subject at a presumed diastolic minimum (left) and systolic maximum (right), as confirmed by the velocity waveform shown for one segment, top. The PIV map is also included for comparison (bottom row). The corresponding video sequence is shown in S5 Video. In the diastolic portion (slower flow), both PIX and PIV methods appear to largely concord. In the systolic portion (fast flow), PIX produces a physiologically plausible output according to the criteria defined above, however the PIV map displays segments containing unexpected oscillations, many of which appear to have much lower than expected velocity (comparable to their diastolic velocity). The maximum velocities reported for PIV are well below those of the PIX algorithm, presumably because the PIV signal has become aliased for many of these vessels at the faster rates of flow experienced during systole. This echoes the observations made for Fig 5 regarding the comparative resilience of PIX to aliasing.

**Fig 7:**
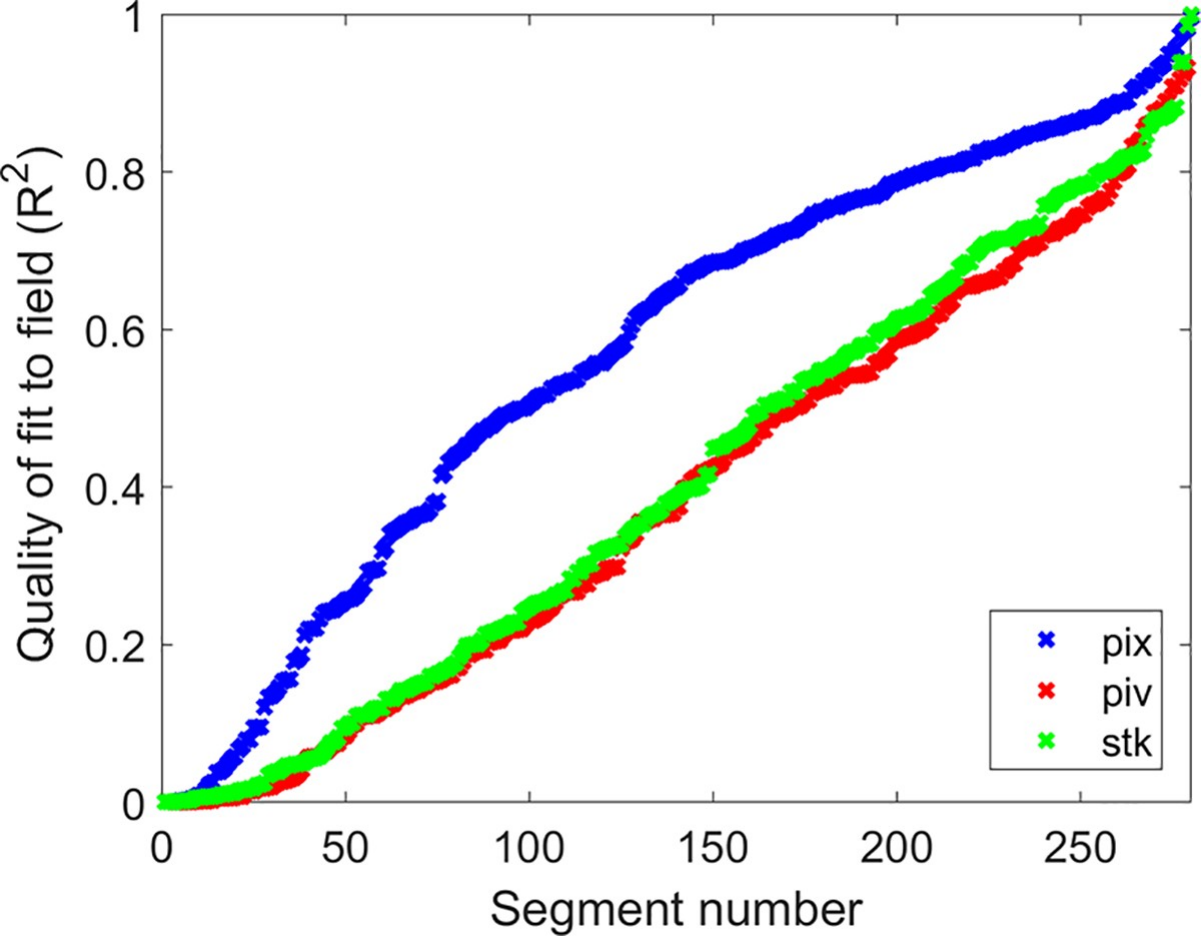
Correlation of velocities measured in each vessel segment to the field average. For each algorithm considered (blue = PIX, green = PIV, red = STK), the average velocity for all visible segments in a field was computed over several cardiac cycles to provide a surrogate for cardiac influence at the capillary level. This procedure was repeated over 7 non-overlapping fields acquired at 300 fps in 2 subjects, yielding a total of 280 unique vessel segments. The goodness of fit (R^2^) to the field average is plotted for each vessel. The higher R^2^ values obtained for the PIX algorithm indicate that it generally returned more physiologically plausible outputs under the imaging parameters used here.

It is difficult to prove that the PIX output should be more physiologically plausible in general, as the true velocity is not known and is liable to vary from the cardiac output according to dynamics events as noted above. Nonetheless, we may expect each vessel to be correlated to the cardiac output to varying degrees, and that any errors in velocimetry will (on the whole) be destructive rather than constructive to this correlation. Taking the average of a large number of segments in each field as indicative of the cardiac output at the capillary level, we calculated the Pearson correlation of each segment in a field to this model. This analysis was repeated over 7 non-overlapping fields in 2 subjects for which we had acquired data over multiple cardiac cycles at 300 fps, for a total of 280 unique vessel segments. The resulting R^2^ of the fit for each segment is plotted in Fig 7 for each algorithm. Values have been sorted in ascending order for ease of visualization. It can be seen that STK and PIV offer similar performance, with stronger correlation evident for PIX across a large number of vessels.

## Discussion

We have presented a new velocimetry algorithm, demonstrated proof of principle and provided example cases which demonstrate its ability to

- provide similar precision and accuracy to manual tracking and to the output of existing approaches (Fig 4)
- track slower, cyclical changes over time reflecting cardiac output (Figs 4–7)
- track rapid changes in time due to transient events such as the passage of white blood cells (Fig 5)
- produce high resolution velocity maps which satisfy physiological expectations regarding constancy of flow within a segment, and abrupt changes before and after junctions (Fig 3, Fig 5, Fig 6)
- render spatial variations along the capillary network with high resolution, including apparent gradients in velocity along some capillary segments (Fig 3, Fig 5)
- produce physiologically consistent measurements even in cases of subjective aliasing of the movie sequence and concomitant non-plausible outputs of other algorithms (Figs 3–7)
- offer superior resilience in the presence of noise (Fig 3)

Whilst existing automated methods (STK and PIV) also satisfied a) and b) above (e.g. Fig 4), the other examples given show clear departures for c) through g). It is important to note that, although not presented here for brevity, we have reviewed a large number of samples acquired from several subjects at varying positions across the retina, and have not observed the opposite pattern in which PIX produced a non-plausible result where the other two did not.

### Spatial distribution of capillary flow

**Fig 8:**
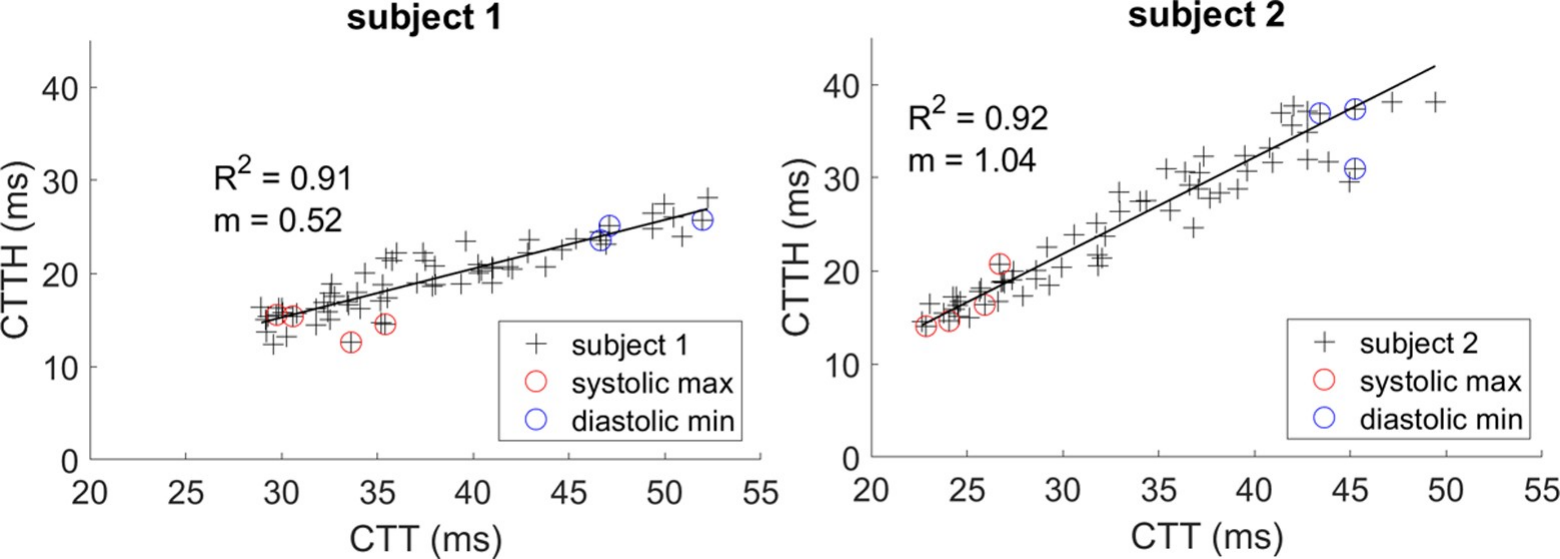
Linear dependence of spatial heterogeneity on mean flow. Sequences correspond to S3 Video and S5 Video. Capillary transit time for individual segments was calculated by dividing segment length by velocity. Network heterogeneity was quantified by the standard deviation (CTTH) and this was plotted against the mean (CTT) for each 100 ms temporal window (symbols). Systolic and diastolic extrema were identified from a representative velocity trace in each field (circles). A strong linear relationship was evident in both subjects, though marked variability in slope exists.

Current understanding of the relationship between capillary flow and oxygen exchange suggests that both overall flow and distribution of flow are important for efficient oxygen delivery [22]. The importance of flow distribution can be marked; if increases in flow are not distributed in a sufficiently equitable manner across the network, oxygen delivery could actually be reduced–such cases have been termed “malignant heterogeneity” [22]. To demonstrate the ability of the proposed algorithm to capture network flow properties in a manner relevant to the modelling of oxygen exchange, we converted measured velocities to capillary transit time (CTT), which is the more appropriate input to such models [22]. CTT is defined as the length of each capillary segment divided by its velocity. We calculated spatial heterogeneity in CTT (CTTH; calculated as the standard deviation of CTT in the imaged field) and overall mean CTT for each 100 ms temporal window for the same subjects shown in Figs 4–6 (S3 Video and S5 Video). The output of this analysis is shown in Fig 8. Both subjects showed strongly linear relationships between CTTH and CTT, with markedly different slopes (m = 0.52 and m = 1.04; R^2^ > 0.9). These slopes are in the estimated non-malignant range (slopes below ~1.3) and agree well with previously published data from a range of species [22]. More subjects will be needed to establish the normative range for this relationship in healthy human retina, however, the present results are of interest to demonstrate the capability of the proposed algorithm to capture these features of network flow, and to highlight the potential for wide variability between individuals, which could in turn influence susceptibility to development of vascular disease.

In addition to differences in flow distribution across the network, some capillaries in the images presented above (e.g. Fig 3, top; Fig 5, longest segment) appeared to show marked gradients in velocity, with smooth and monotonic variation in flow along the length. To our knowledge this phenomenon has not been reported previously. It is possible for such changes to result at least in part from a change in vessel trajectory in the axial direction, however, the fact that they are rendered by the proposed algorithm speaks to its high spatial fidelity.

### Aliasing and noise

In cases where the flow is subjectively ambiguous in a vessel (e.g. due to aliasing or due to low contrast), it is not impossible to judge which algorithm (if any) has produced the correct output. The criterion that we have adopted is one of physiological plausibility, hence we have pointed above to the faithful replication of the expected cardiac cycle across time, contiguity of the spatial map within a segment, and demarcation of sudden changes between segments to infer the appropriateness of the PIX output. These features are particularly compelling because there is no spatial or temporal smoothing applied to the raw data. Overall, these considerations lead us to believe that the PIX method may offer reduced sensitivity to noise and aliasing compared to other methods, especially in regards to the extraction of periodic variations in the data (sudden discontinuities seen in the other methods are not evident with PIX). Such features are highly desirable for practical application to the study of large numbers of capillary segments and in clinical populations which may feature aberrant flow profiles, increased scatter, require the use of longer imaging wavelength or more peripheral imaging location (with commensurately lower contrast), or yield fewer frames in a given area due to poorly controlled eye movements.

The potential for PIX to avoid aliasing at lower frame rates allows imaging over an extended field with present adaptive optics imaging hardware. In our system, the frame rate is limited by the number of pixel rows which must be read out in order to constitute a frame; hence a faster frame rate must be accompanied by a shorter field height. Raster based systems possess analogous limitations [15, 19]. However, a shorter field height allows only a smaller portion of the vascular network to be imaged which greatly diminishes the efficiency of data collection and observation of natural flow patterns across the network. This problem becomes exacerbated with longer observation times as natural eye movements shift the imaged field. Hence, the ability to operate at slower frame rates is highly desirable.

### Phantom data

In the past we have resolved questions regarding aliasing by the use of phantom data [4]. Although not shown here, a simple model comprised of identical cells separated by identical periods is actually handled poorly by PIX. This is because the “cells” will correlate just as easily with any other cell as they do with themselves. This does not inherently pose a problem for the PIV or STK algorithms (subject to the velocity remaining within sampling constraints). PIX seemingly requires nuanced differences to be associated with members of the passing cell train, which limits the ability to assess it against a simple phantom model, but on the other hand probably underlies the surprising resilience to aliasing that we have shown above. It would be possible to develop a more realistic phantom model, but we have elected not to pursue this further given the need to incorporate the poorly understood image formation properties of blood cells and vessels walls [18], physiological variability in the period between succeeding cells, etc.

### Processing speed

The relative processing speed of each approach depends on the size of the region of interest and any strategies employed to ignore areas devoid of flow information. In general, the number of correlations computed with PIV scales with the width of the region of interest (w) as ~ w^2^, whereas PIX scales as ~w^4^ because each additional pixel must be compared with all other pixels. However, individual correlations are computed much more quickly for PIX (e.g. one correlation of two 30-element vectors, compared with 30 correlations of successive image ROI’s), such that the faster method depends on the absolute size of the image. As an example, for the sequence underlying Figs 4 and 5, PIX and PIV processing speeds were almost identical and some 5 times slower than the STK method; if correlations are not limited to pixels underlying the vascular mask, PIX is approximately 5 times slower than PIV. It should be noted that both PIX and PIV are highly parallelizable and, although we did not make use of parallel processing here, we expect similar gains for each from parallelization.

### Removal of background structure

A useful step in the PIX algorithm was to subtract the standard deviation of each pixel from the RMS error, which was seen to effectively remove the background structure from the similarity image (Fig 2D). Why should this occur? For a non-correlated pair of pixels, the expected value for mean square error is proportional to the sum of their variances. One of these variance terms is common to all pixels in the similarity image, because it represents the reference pixel whose velocity is to be determined. Therefore the salient differences between most of the pixels in the similarity image occur largely due to their own variance, which can be subtracted away to equalize the relative contrast between all pixels. Of course, the expected error for pixels which are actually correlated to the reference pixel cannot be approximated in this way; after the normalization procedure just described, these pixels now appear bright against the relatively constant background of non-correlated pixels.

### Limitations

**Fig 9:**
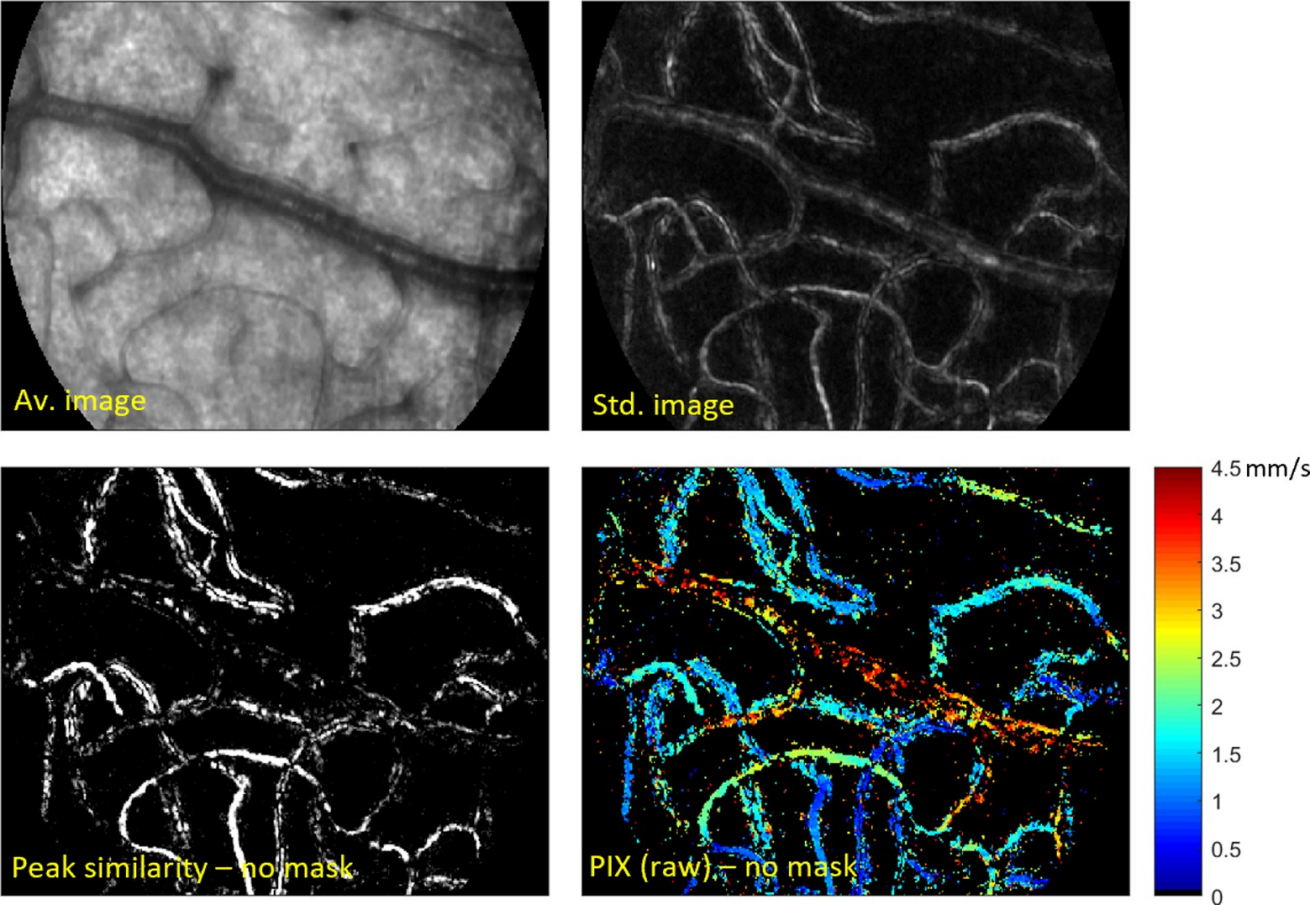
Co-localization of high reliability of velocimetry with the vascular tree, but not necessarily with motion contrast. Video sequence acquired at 200 fps is shown in S6 Video. Top left: average image. Top right: standard deviation image (motion contrast or perfusion image). Bottom left: Peak similarity measure (derived as illustrated in Fig 2) obtained for each pixel, plotted on log scale, without any binary masking of the vascular tree. Bottom right: Velocity map produced from this sequence without any binary masking of the vascular tree. Scale bars show 50 μm.

The proposed method does not necessarily return a reliable velocity estimate for each perfused pixel (i.e. for all bright pixels in the motion contrast image). However, it does return a metric which can be used to assess the quality of flow information, which often proves difficult with other methods. This is achieved by consideration of the height of the peak on the normalized similarity map (e.g. the intensity of the highlighted pixels in Fig 2D). Fig 9 allows comparison of areas of absorption contrast (top left) or motion contrast (top right) with the normalized similarity metric (bottom left) and resulting velocity map (bottom right). To aid visualization the peak similarity image has been scaled to saturate for values > 10 σ. Perhaps contrary to expectations, the velocimetry quality statistic does not appear easily predicted from the motion contrast image (compare for example the sporadic bright patches along the central large arteriole for the peak similarity image, which are not evident in the motion contrast image). In other words, some vessels may show high variance yet do not yield a high proportion of pixels suitable for analysis. The similarity metric ensures that only those areas of the image which do produce a reliable signal are used in the final velocity map. The appropriateness of this metric is re-enforced by calculation of the corresponding velocity map without the use of any binary vessel mask (bottom right), which shows that the vascular tree is well reconstructed.

There are many potential reasons for areas producing high “motion contrast” to provide sub-optimal flow information. The sequence shown in Fig 9 was acquired at 200 fps which is well below the sampling speed required to image faster capillaries with other velocity approaches [19], let alone the larger arteriole stretching across the frame from right to left. However, the regions that do satisfy the reliability criterion appear to match physiological requirements noted above i.e. contiguity within segments and sharp differences between segments. Aside from temporal sampling limitations, other reasons for poor signal correlation despite high flow-related variance could include proximity to the image edge, proximity to branch points, depth-based alteration and associated inversion of contrast [18], vessels diving out of the plane, variable thickness of the vessel wall, differences in available pixel data and image quality due to eye movements, or spatial variations in optical quality of capillaries (the “windowing” phenomenon suggested above).

When combining the measured forward- and backward-shifted velocities for each pixel, we elected to average the two measures to improve robustness. A potential downside of this approach is that for many pixels in the image there is only one direction in time which should be considered. For example, a pixel close to but downstream of an arteriolar branch should consider only correlations that are forwards in time, else it will look upstream and “dilute” its velocity with that of the parent segment. Similarly, the parent segment should only look upstream to avoid contaminating its measurement with those of its daughters. Other examples could include pixels near the image edge, those which send/receive blood to/from an out-of-plane “diving” vessel, or pixels near an area in which flow information is otherwise lost due to poor image quality or pathological flow, e.g. a microaneurysm.

### Improvements

It may be possible to extend the simple, individual pixel method advocated here to exploit the necessary similarity in sign and direction of velocity that must exist between neighbouring pixels on the vascular tree. Initial values returned by the proposed algorithm could be used to seed the consideration of blocks (as in PIV) and/or lines (as in STK) of related pixels, or to aid the tracking of individual particles.

Results presented here were obtained by shifting a single frame forwards or back in time. This is necessary to capture fast flow in short segments (for example, a cell flowing at 4.5 mm/sec will traverse 15 microns between 2 frames at 300 fps). However, results obtained in slower and longer vessels could be obtained equally well by considering longer frame delays. It may be possible to conduct a multi-scale analysis with frame lags of differing sizes to improve robustness (at the expense of processing time).

It would not be possible, on the other hand, to consider *sub*-frame lags, because (at presently available frame rates) cells generally move far too great a distance between successive frames to allow sensible interpolation of data. However, sub-pixel determination of the “most similar” pixel in the similarity maps should be possible since the correct pixel is typically enclosed in a “cloud” of bright pixels (e.g. Fig 2F).

### Extension to other imaging modalities

The above considerations leave the possible extension of the algorithm to other applications beyond retinal imaging unclear, but suggest that the greatest success will be encountered for data in which the moving elements and or/their patterns of arrangement carry some sort of unique “signature”, i.e. are distinguishable in some way from their neighbours.

Within the field of retinal imaging, although we have presented the PIX method using data acquired from a flood-based system, there is no reason in principle that the method should not be applicable to raster systems. The method does require a stable relationship to be established between two pixels over an extended period, which is made more difficult in the presence of image distortion seen by scanning systems as a result of the perpetual motion of the eye. This issue can theoretically be dealt with in post-processing without any changes to existing hardware [24].

## Supporting information

S1 Demonstration Code and example data. Data file consists of 2 scaling parameters (‘fps’ and ‘pixel_diam_mm’), a binary vascular mask (‘maskIm’), and 30 frames of image data (‘windowArray’) which represents the 100 ms example epoch. If the files “pix_demo.m” and “demo_data.mat” are on the MATLAB path, the example can be run by executing “pix_demo.m” as a script from within MATLAB. A colour map similar to those shown in the paper is the expected output.”
(ZIP)

S1 Video Example sequence of high quality. Data acquired at 400 fps and corresponds to Fig 1 (top), Fig 2, Fig 3 (top). Top panel of video shows raw frames and bottom shows the same frames after temporal filtering described in text.
(AVI)

S2 Video Example sequence of low quality. Data acquired at acquired at 400 fps and corresponds to Fig 1 (bottom) and Fig 3 (bottom). Top panel of video shows raw frames and bottom shows the same frames after temporal filtering described in text.
(AVI)

S3 Video Longer example sequence showing variation across the cardiac cycle. Data acquired at 300 fps and corresponds to Fig 4, Fig 5 and Fig 8 (left). Top shows raw data and bottom shows the same frames after temporal filtering described in text.
(AVI)

S4 Video Evolution of velocity map over time. Velocity maps were calculated for every third frame of S3 Video. Top shows raw maps and bottom shows the same data after spatial filling described in the text.
(AVI)

S5 Video Longer example sequence showing variation across the cardiac cycle. Data acquired at 300 fps and corresponds to Fig 6 and Fig 8 (right). Top shows raw data and bottom shows the same frames after temporal filtering described in text.
(AVI)

S6 Video High quality example sequence used to compare motion contrast with reliability of velocimetry. Data acquired at 200 fps and corresponds to Fig 9. Top shows raw data and bottom shows the same frames after temporal filtering described in text.
(AVI)
